# Targeting secretory autophagy in solid cancers: mechanisms, immune regulation and clinical insights

**DOI:** 10.1186/s40164-025-00603-0

**Published:** 2025-02-01

**Authors:** Xinyu Li, Haiying Zhao

**Affiliations:** Department of General Surgery, Fourth Affiliated Hospital of China Medical University, Shenyang City, 110032 Liaoning Province China

## Abstract

Secretory autophagy is a classical form of unconventional secretion that integrates autophagy with the secretory process, relying on highly conserved autophagy-related molecules and playing a critical role in tumor progression and treatment resistance. Traditional autophagy is responsible for degrading intracellular substances by fusing autophagosomes with lysosomes. However, secretory autophagy uses autophagy signaling to mediate the secretion of specific substances and regulate the tumor microenvironment (TME). Cytoplasmic substances are preferentially secreted rather than directed toward lysosomal degradation, involving various selective mechanisms. Moreover, substances released by secretory autophagy convey biological signals to the TME, inducing immune dysregulation and contributing to drug resistance. Therefore, elucidating the mechanisms underlying secretory autophagy is essential for improving clinical treatments. This review systematically summarizes current knowledge of secretory autophagy, from initiation to secretion, considering inter-tumor heterogeneity, explores its role across different tumor types. Furthermore, it proposes future research directions and highlights unresolved clinical challenges.

## Introduction

Macroautophagy, often referred to as autophagy, is a highly conserved regulatory mechanism responsible for degrading cellular substances to recycle energy and materials [1]. Most organisms/cells rely on autophagy to maintain metabolic processes. For example, autophagy activation promotes the clearance of misfolded proteins associated with neurodegeneration [2]. The effective strategies of clinical treatment targeting autophagic mechanisms are fraught with complexities and associated with tumor stage, biology, and the surrounding microenvironment [3]. As an intracellular dynamic process, it accepted autophagy-related genes (ATGs) regulation to finish the formation of two-membrane vesicles (autophagosomes) and encapsulate cellular cargos to fuse with lysosomes, leading to cargos degradation in assistance with hydrolase in the lysosomes [4]. In detail, when energy or metabolic intermediates are insufficient, rapamycin complex 1 (mTORC1) senses metabolic imbalances. This activates unc-51-like kinase (ULK) complexes, including ULK1, ULK2, focal adhesion kinase family-interacting protein of 200 kDa (FIP200), ATG13, and ATG101, to initiate autophagy and regulate autophagosome formation [5, 6]. Activation of ULK complex catalyzes phosphatidylinositol-3-phosphate (PI3P) production *via* autophagy-specific vacuolar protein sorting 34 (VPS34) complex I (including VPS34, Beclin-1, ATG14, and VPS15), PI3P located on autophagosome membrane triggers the recruitment of ATGs [7, 8]. Initiation and maturation of autophagosome need ATG8 family proteins (microtubule-associated proteins 1 A/1B-light chain 3 (LC3) and GABA(A) receptor-associated protein (GABARAP)) lipid coupling in assistance of ATG16L1-ATG5-ATG12 complex, ATG3, and ATG7 [9, 10]. Under nutrient-deficient conditions, autophagosome cargo loading can occur through non-selective or selective mechanisms, with passive selection dependent on autophagy cargo receptors (ACRs) [11]. In addition to classical ATG-mediated autophagy, chaperone-assisted autophagy, involving chaperone-mediated activity and lysosomal membrane invagination, also contributes to the degradation of cellular materials [1, 12]. Due to the extensive membrane distribution of ATGs and its effect on membrane structure elongation and maturation, ATGs are also associated with cell endocytosis, cytokine secretion [13, 14], phagocytosis [15, 16], intracellular vesicle transport [17–19] and exosome secretion [20–22]. The release of specific substances mediated by ATGs or autophagosomes, known as secretory autophagy, facilitates the autophagy-related secretion process [23, 24]. Secretory autophagy delivers functional payloads to the tumor microenvironment (TME), mediating intercellular communication and TME remodeling. Understanding the mechanisms of secretory autophagy, including cargo selection and substance release, is essential for elucidating its role in tumor progression. This review highlights the biological processes (cargo selection, substance release) underpinning secretory autophagy, emphasizing its function and significance within the TME. Advancing knowledge in this field has the potential to inform the development of novel anti-tumor therapies.

## Secretory autophagy: an unconventional protein secretion

Large granules labeled with Golgi reassembly and stacking protein (GRASP), located near the endoplasmic reticulum exit, are recognized by ATGs, which gradually induce secretory autophagosome membrane elongation and maturation [25–27]. Secretory autophagosomes can directly fuse with the plasma membrane to release packaged substances or fuse with the multivesicular body (MVB) to form amphisomes. Amphisome fuses with the plasma membrane to release the encapsulated substances outside the cell [25–27]. Studies in yeast have demonstrated that Acb1 secretion depends on ATGs-mediated autophagosome formation and GRASP involvement (Grh1 in yeast) [25, 28]. In mammalian cells, secretory autophagy of IL-1β is dependent on GRASP and RAB8a-mediated vectorial sorting to plasma membrane [26]. RAB8a has been found to regulate the secretory autophagy process of alpha-synuclein in the nervous system [27]. Inhibition of autophagy flux using the pharmacological inhibitor 3-methyladenine (3-MA) or ATG5-targeting siRNA significantly increased the secretory autophagy of α-synuclein [27]. Moreover, inhibition of the fusion of autophagosomes and lysosomes by bafilomycin A1 (BafA1) also promoted the secretion of α-synuclein rather than degradation [27]. Increased microtubule acetylation, induced by histone deacetylase 6 (HDAC6) down-regulation, reduces autophagosome-lysosome fusion and enhances α-synuclein secretion [27]. Inhibiting the fusion of autophagosome and lysosome or changing the acetylation level of microtubules will change the kinetics of the autophagosome, leading to its switch to the secretory pathway. The soluble *N*-ethylmaleimide-sensitive factor attachment protein receptor (SNARE) family plays a key role in plasma membrane fusion. Plasma membrane SNARE (SSO1) is essential for secretory autophagy-mediated membrane fusion, whereas degradation-associated fusion relies on the SNARE complex (vesicle-associated membrane protein [VAM]3/7) [25]. Similarly, RAB8a acts as a regulator of polarization sorting into the plasma membrane [25, 28], while RAB8b regulates the maturation of degrading autophagosomes [29]. Autophagosome fuses with MVB to form amphisome, transferring cargos to plasma membrane/lysosomes to determine the destination of transport.

## Autophagy and secretory autophagy differences

Classical autophagy and secretory autophagy share some similarities but also show distinct differences in their mechanisms and functions. For example, both require the formation of separate vesicles from the endoplasmic reticulum, but secretory autophagy can also occur in the absence of the ULK complex [30]. The vesicles in the secretory autophagy pathway contain more proteins involved in plasma membrane fusion compared to those associated with lysosome fusion, highlighting a key distinction between the two pathways [25]. Both are highly dependent on ATGs, and some ATGs play dual regulatory roles, such as ATG5 [20]. For example, in the classical autophagy pathway, ATG5, ATG12, and ATG16L form a functional complex to promote LC3I to LC3II conversion, thereby promoting autophagosome elongation. In the secretory autophagy pathway, ATG5 maintains the acidification of secretory vesicles, contributing to substance secretion [20, 31]. Further, LC3 performs distinct functions in the two pathways. In classical autophagy, LC3 mediates the elongation and closure of autophagosomes, whereas, in secretory autophagy, it facilitates the sorting of substances, particularly RNA and RNA-binding proteins (RBPs) [32]. This topic is explored in greater detail in the following sections.

### Origin differences

The omega-shaped domains (omegasomes) of the endoplasmic reticulum (ER) are considered primary contributors to autophagosome biogenesis, although their exact function remains unknown [33]. Secretory autophagy is partly derived from a relatively special ER-derived precursor structure (unconventional protein secretion compartments (CUPS)), which is similar to the mammalian omegasome [34, 35]. Studies have shown that classical autophagy inducers facilitate the simultaneous formation of CUPS and omegasomes, as both express PI3P and ATGs [34, 35]. However, whether these represent the same structure requires further investigation. Classical autophagy usually requires the involvement of the ULK1 complex to promote isolation membrane formation from the omegasome [36, 37]. ATG9 and ZFYVE1/DFCP1 are recruited to the omegasome to facilitate vesicle biogenesis and elongation during classical autophagy [31]. In noncanonical autophagy, *de novo* autophagosome synthesis is unnecessary; instead, vesicle recycling facilitates the transport of substances [30]. This provides new insights into the vesicular origin of secretory autophagy versus conventional autophagy. Omegasomes are characterized by the dual FYVE domain protein ZFYVE1/DFCP1. While depletion of ZFYVE1/DFCP1 does not prevent starvation-induced autophagy, amino acid starvation, a classical omegasome inducer, may explain this outcome. ZFYVE1/DFCP1 appears to affect contraction only when omegasome diameters are sufficiently large. Although ZFYVE1/DFCP1 does not impact macroautophagy, it inhibits selective autophagy of mitochondria, protein aggregates, and micronuclei [33, 38]. These findings provide insights into the distinct molecular mechanisms underlying selective autophagy and macroautophagy, suggesting that different signaling pathways mediate these processes. It is worth noting that ATP binding and hydrolysis control the multimerization state of ZFYVE1/DFCP1, thereby driving the large omegasome to shrink and become smaller by mechano-chemical energy [33, 38]. This suggests that secretory autophagy may also be dependent on the hydrolysis of ATP by ZFYVE1/DFCP1. However, the specific mechanisms of both are still unclear and need to be further determined.

**Fig. 1:**
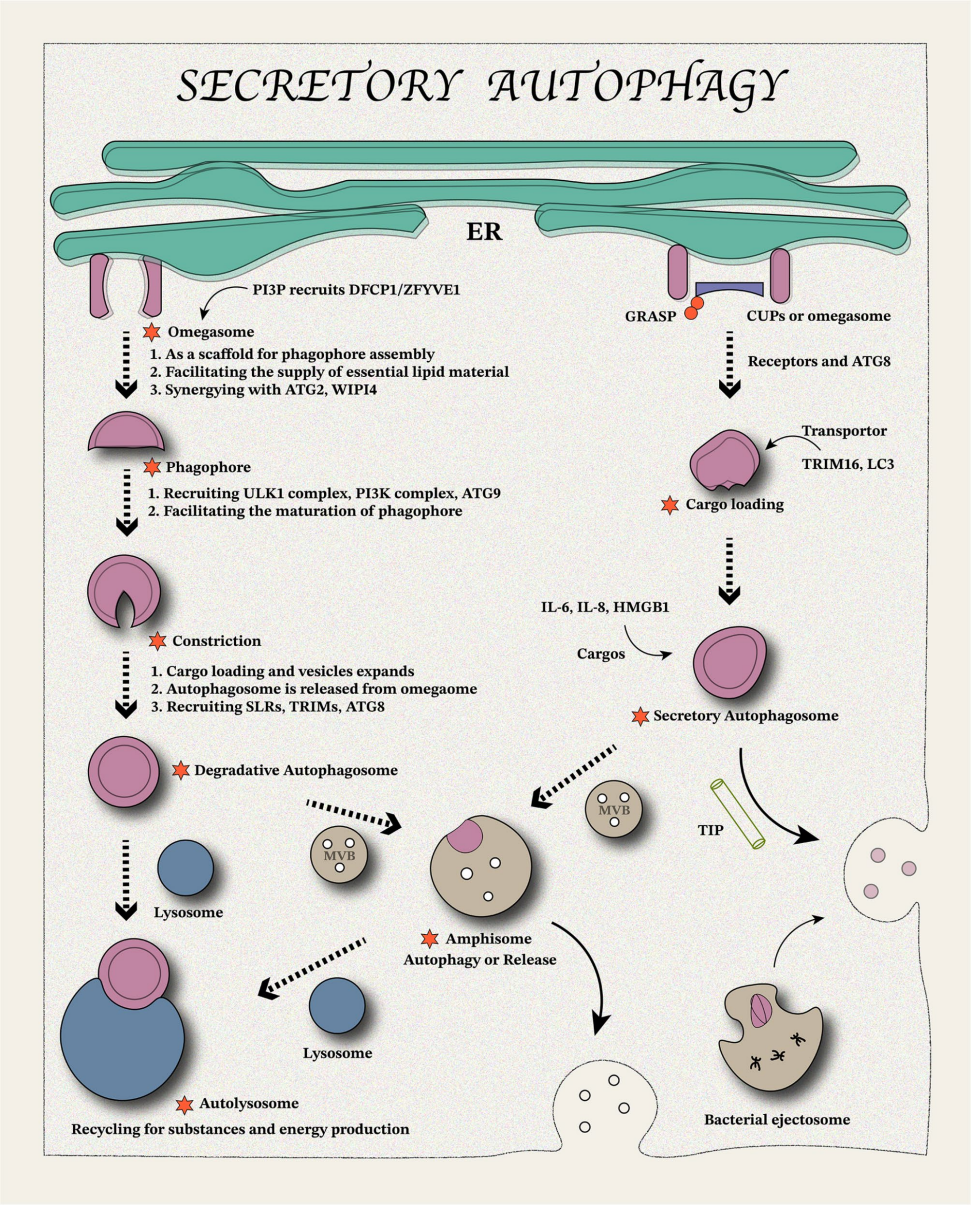
Mechanisms of secretory autophagy and interactions with classical autophagy

CUPS are membrane-like structures that are thought to contain some specific Golgi-associated proteins, for example, Grh1 (yeast equivalent of GRASP), Bug1, the membrane tethering factor Uso1, the t-SNARE Sed5, Sec7, and Pik1 [39]. Moreover, the SNARE protein (SSO1) expression required for plasma membrane fusion is critical for secretory autophagy-derived vesicles but independent of traditional autophagy SNARE complex (VAM3/VAM7)-related degradation [25]. Vacuolar protein-sorting 34 (VPS34) as a phosphatidylinositol 3-kinase is needed for maintaining the biological functions of CUPS [39]. It is worth mentioning that VPS34 functions as a key regulator involved in vesicle release, as well as the SNARE family [39–44]. This, in part, suggests that part of SNARE family proteins can be recruited during the formation of secretory autophagic vesicles to provide a basis for subsequent plasma membrane secretion. RAB8 is essential for vesicle trafficking to the plasma membrane, a feature that distinguishes secretory autophagy from classical autophagy [26, 27]. Differences in specific protein expression and recruitment likely drive the distinct vesicle transport pathways that separate secretory autophagy from traditional autophagy (Fig. 1).

### Cargo differences

#### TRIM16-mediated cargo selection

**Fig. 2:**
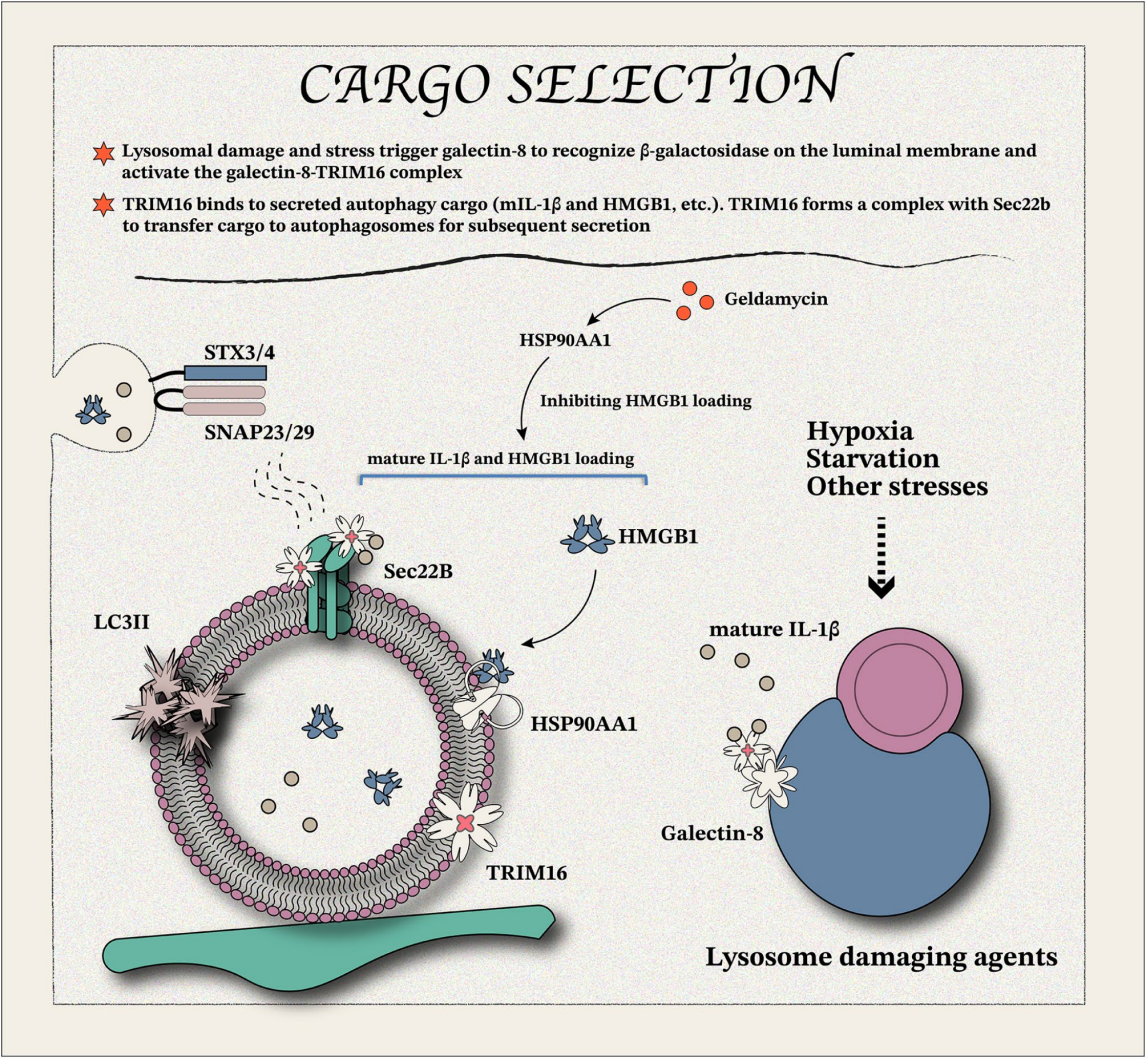
Secretory autophagy: cargo selection

The nuclear protein high mobility group box-1 (HMGB1) is secreted in response to stress stimuli, suggesting its role as a danger-associated molecular pattern [45–47]. Lacking an ER-targeting signal peptide, HMGB1 is secreted *via* an unconventional, ER-Golgi-independent pathway [48–50]. Studies have found that heat shock protein 90α family class A member 1 (HSP90AA1) acts as a stress-related protein, which can regulate the transport of HMGB1 from the nucleus to the cytoplasm by direct binding [51]. Geldamycin, an inhibitor of HSP90AA1, blocks HMGB1 secretion by disrupting its intrinsic trafficking signal. Similarly, HMGB1 secretion is strongly inhibited during secretory autophagy when early autophagy inhibitors or ATG5 knockouts are employed [51]. This suggests that differences between secretory and conventional autophagy may depend primarily on substrate-specific structural engagement or functional regulation (Fig. 2). Secretory cargos typically lack endoplasmic reticulum targeting signal peptides and are secreted through unconventional pathways. These substances may exhibit substrate-specific secretion mechanisms. Further research is needed to clarify the mechanisms of cargo selection and to determine whether substrate selectivity is active or passive. It is worth noting that viruses and insulin can also be released through secretory autophagy at baseline housekeeping levels [52, 53]. The biological significance and mechanisms of cargo sorting in secretory autophagy are not fully understood, but current evidence suggests a role for ubiquitination and TRIM-mediated selectivity [54, 55]. Tripartite motif-containing protein 16 (TRIM16) interacts with IL-1β [56], and its absence significantly reduces the interaction between IL-1β and LC3, as well as the interaction between Sec22b and TRIM16^Δ373–564^. This impairs IL-1β autophagy-dependent secretion, which requires ATG5 and Galectin-8 [57]. Sec22b knockdown does not affect autophagosome formation or autolysosome function. However, reduced Sec22b levels impair IL-1β autophagy-dependent secretion independently of ATG9, Syntaxin 17 (responsible for autophagosome-lysosome fusion), and p62 [57]. Based on the function of TRIM16, IL-1β autophagy and secretory autophagy appear to share common early-stage mechanisms. However, their distinct outcomes are determined during the middle and late regulatory stages. Galectin family members should be considerable in the cargo selectivity because of the recognition of β-(1–4) linked galactosides [58–61]. Serum ferritin, another unconventional secreted protein associated with inflammation, has also been shown to be secreted through unconventional pathways [62]. TRIM16, galectin-8 (but not galectin-3), and Sec22b (but not syntaxin 17) mediated secretory autophagy [57]. However, TRIM16 cross-links with the p62-KEAP1-NRF2 complex under oxidative and proteotoxic stress conditions to promote autophagic degradation of misfolded proteins [63]. TRIM16-mediated protein degradation may require oxidative and proteotoxic stress environments as well as protein misfolding, while for functionally/conventionally secreted proteins, TRIM16 may play a cargo-loading function. Cargo selection provides an optional termination (lysosome or exportation) in the autophagosome-related process to enable the exportation of leaderless cytosolic substances from intracellular metabolism. Different substrates may be selected differently, depending on the structure and function of the protein, peptide, or vesicle itself.

#### LC3-dependent extracellular vesicle (EV) loading

**Fig. 3:**
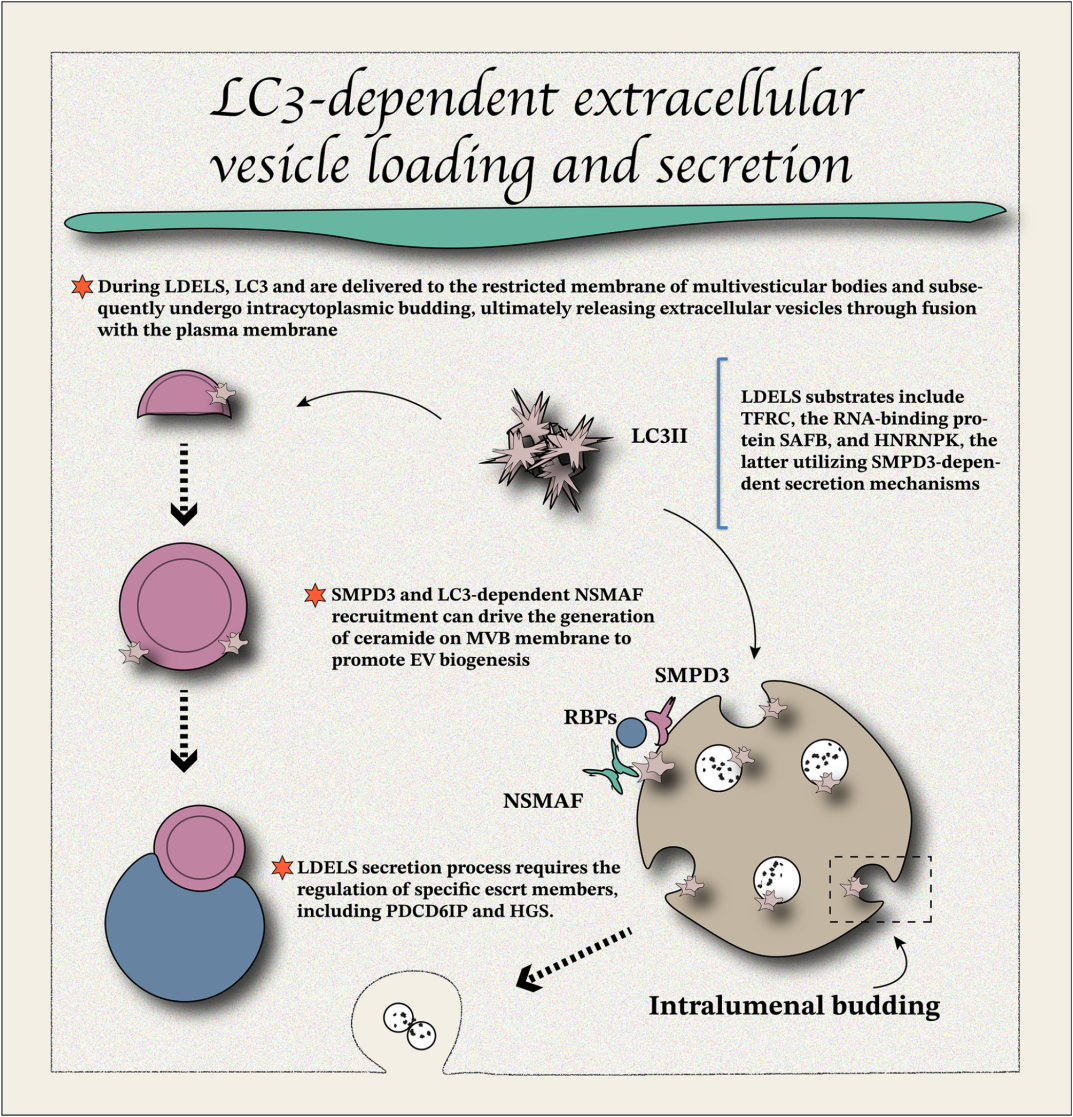
The mechanisms of LDELS

Debnath et al. described the LC3-dependent EV loading and secretion (LDELS) mechanism for RNA and RBPs, which relies on the LC3-conjugation machinery (Fig. 3) and requires the recruitment of nSMase2 and Fanconi anemia-associated nuclease (FAN) [32]. Ceramides produced by nSMase2 may play a functionally unique membrane regulation role (negative membrane curvature) by driving membrane microdomain formation, leading to endosomal sorting complex required for the transport (ESCRT)-independent luminal budding at MVB-restricted membranes [32]. Unlike nSMase2, FAN cannot be detected on EVs, indicating that FAN may only transiently interact with LC3 or act on endoplasmic reticulum/Golgi apparatus to participate in the secretory process. Recent studies have outlined inter-organelle communication in MVB cargo sorting, providing new insights into the relationship between FAN and secretory autophagy [64, 65]. As an F-actin binding protein, FAN contributes to cytoskeleton reorganization, which reveals the critical role of FAN in membrane transport and formation during the LDELS process [66]. LC3 selectivity for cargos may be primarily restricted to proteins or RNAs through specific structural motifs. In general, the structures of ATG8 family proteins contain functional domains that interact with RBPs (LIR motif), and mutations in the LIR motif are sufficient to impair EV secretion [67]. This aligns with the observation that many LDELS-related molecules are positively expressed in RNA delivery particles, such as stress granules and P-bodies [68, 69]. A recent original study has reported a previously unrecognized protein named transferrin receptor (TFRC), which acts as a specific integral membrane protein. Its secretion is strongly dependent on the binding sites with ATG8 family proteins but not other essential ATGs for autophagosome formation. Mechanistically, ATG8 family proteins directly bind to the cytoplasmic domain of TFRC (LIR motif) and selectively load TFRC into EVs [70]. EV-TFRC secretion requires the participation of ESCRT components and the small GTPase RAB27a [70], which is extremely similar to the traditional EV secretion process [71–75]. It is implied that TFRC secretion and traditional EV secretion processes possess unified regulatory pathways. This suggests the critical role of ATG8 family proteins in secretory autophagy, as well as non-classical autophagy processes.

This observation aligns with the non-classical autophagy pathway described earlier. While classical autophagy relies on the ATG8 protein family binding to lipids, such as phosphatidylethanolamine (PE), to mediate autophagy, the non-classical autophagy pathway is primarily mediated by the conjugation of ATG8s to single membranes (CASM), including late endosomes and lysosomes [30]. Unlike classical autophagy, the non-classical pathway does not depend on *de novo* autophagosome synthesis but requires ATG8 lipidation. This highlights that the regulation of ATG8 by proteins such as ATG3, ATG4, ATG5, ATG7, ATG10, ATG12, and ATG16L1 is consistent with the mechanisms of classical autophagy [30]. Interestingly, an increased ATG8-PS/ATG8-PE ratio has been observed in the CASM pathway, suggesting that ATG8-PS may serve as a potential marker of non-classical autophagy [30]. Although secretory autophagy shares molecular regulatory features with CASM and classical autophagy, the precise relationship between these pathways requires further investigation.

### Vesicle transport differences

RAB37 GTPase regulates the exocytosis of intracellular cargo by binding to the GDP/GTP form. RAB37 can interfere with the activation of autophagy and the secretion of tissue inhibitors of metalloproteinase-1 (TIMP1) in lung cancer cells under starvation conditions. As a motile protein, RAB37 hydrolyzates GTP to provide sufficient energy for material transport and subsequently participates in the fusion of vesicles and plasma membranes in a Sec22B-dependent manner, thereby regulating the secretion of TIMP1 [76, 77]. It has been reported that a novel autophagy inducer, niclosamide (an anti-helminth drug), significantly increased autophagic flux and promoted insulin secretion through an autophagy-dependent secretion pathway with the involvement of RAB37, which improved blood insulin and glucose concentrations [53]. RAB37 is also involved in regulating autophagy, suggesting that secretory autophagy and conventional autophagy have many overlapping regulatory mechanisms, but SNARE family proteins, especially Sec22B, may be the key factors.

Multiple studies have demonstrated that mATG8 proteins and their coupling systems are not essential for autophagosome formation [78, 79]. Unlike the traditional autophagy pathway, this process occurs independently of ATG7. Interestingly, RAB7, a key protein responsible for autophagosome-lysosome fusion, has been shown to regulate secretory autophagy. Knockdown of RAB7 increases secretory autophagy, while knockdown of SNAP23 inhibits this process [80–82]. SNAP23, a well-characterized regulator of vesicle-plasma membrane fusion, is critical for the fusion process during secretory autophagy [40, 83]. RAB7, an endosomal protein, primarily regulates the fusion of autophagosomes with lysosomes [84–87]. Furthermore, RAB7 knockdown may interfere with the early stages of exosome formation. In autophagosome membranes and other single-membrane organelles, mATG8 lipidation is required for the formation of single-membrane structures. This process involves recruiting ATG12, ATG7, and ATG10. With the persistent activity of ATG7 and ATG10, the ATG5-ATG12 complex associates with ATG16L1, leading to ATG8 conjugation (atg8ylation) [88, 89]. This conjugation facilitates cargo sequestration within autophagosomes [90], membrane remodeling [91], structural perturbation [92], and membrane expansion. These mechanisms collectively contribute to the repair and maintenance of single-membrane organelles.

## Status of secretory autophagy in tumour progression

Secretory molecules act as critical information carriers, transmitting biological signals within the TME [93–96]. These molecules have been recognized for their potential in prognosis evaluation and early diagnosis of various tumors [97–99]. Autophagy has traditionally been characterized as a cellular mechanism essential for degrading damaged organelles and misfolded proteins, providing materials and energy for cellular reuse [3, 100, 101]. However, further investigation shows that autophagy has two distinct pathways: one involves delivering cargo to lysosomes for degradation, while the other transports cargo to the cell membrane for secretion [20]. This autophagy-dependent secretory process plays an essential role in both physiological and pathological cellular metabolism, including processes such as insulin secretion, mitochondrial clearance, and the secretion of HMGB1 protein [51, 53, 82]. Given these dual roles, a critical question arises about the significance of secretory autophagy in cancer cells.

**Fig. 4:**
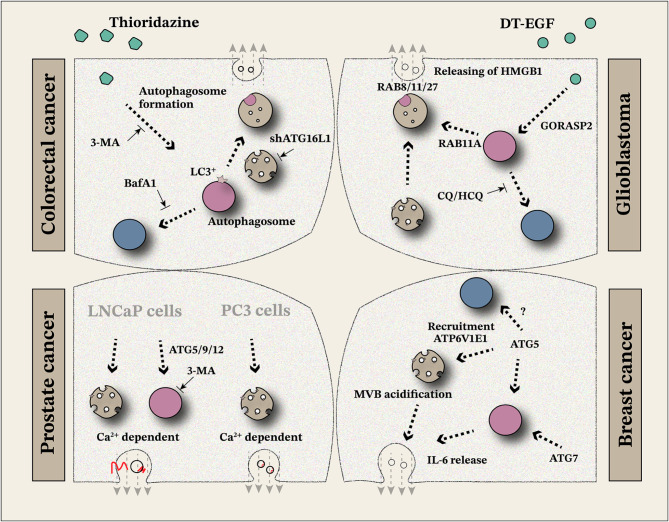
Regulation of secretory autophagy in tumour progression

Excessive activation of autophagy is a classic characteristic of various solid tumors, and in-depth basic exploration and clinical trials have been conducted to target autophagy [1, 102]. Abnormally high expression of ATGs contributes to the malignant progression of solid tumors by enhancing baseline autophagy or regulating organelle acidification [103–105]. ATG5 specifically decreases late endosome acidification by removing ATP6V1E1, leading to a shift from autophagic degradation to secretory process [20]. Almost all solid tumors highly express ATG5, ATG7, ATG6L1, and secretion-related genes such as RABs [100, 106–108], revealing the interaction between tumor progression and intracellular cargos transport. Cells with a high level of autophagy release more secretory substances and exhibit exosome heterogeneity to a certain extent [109]. Theoretically, exosomes can carry ATGs and RABs, and the distribution of these proteins in exosomes also provides more options for cargo loading. A better understanding of protein conformation and molecular docking may provide a basis for precise treatment. To survive, tumor cells increase their metabolism to produce more intermediate metabolites/energy and accelerate internal circulation [110–113]. When excess metabolic byproducts exceed the cell’s digestive capacity, they are expelled through the secretory pathway, highlighting the requirement of secretory autophagy, which couples autophagy to secretion (Table 1) [20, 109, 114–120]. Moreover, high mutation rates in oncogenes such as *TP53* and *KRAS* [121–124] may induce structural changes in proteins, forcing alterations in secretion pathways. These changes contribute to the enhanced survival of tumor cells under stress conditions. Given the heterogeneity of tumors, this study categorizes different solid tumors to elucidate the mechanisms, significance, and potential exploration strategies for secretory autophagy in tumor progression (Fig. 4).

**Table 1:** The function of secretory autophagy-related molecules released by solid tumour cells

	Cell types	In vivo/In vitro	Inducer	Mechanisms	Effection	PMID
Colorectal cancer	HT29, LoVo, HCT116, and RKO	In vivo and In vitro	Thioridazine	——	Immunogenic cell death	[119]
Colorectal cancer	HCT116	In vitro	shATG16L1	——	Exosome decreasing	[20]
Glioblastoma	U251, LN229, Primary human GB1 and GB2	In vivo and In vitro	DT-EGF	HMGB1/RAGE/ERK/IKB/NF-kB	Promoting immunogenicity and M1-like polarization of TAM	[118]
Prostate cancer	LNCaP, PC3	In vivo and In vitro	Cell membrane invaginated	CAV1 and Ca2 + influx	Increasing malignant ability	[114]
Breast cancer	MDA-MB-231, MCF7 and 4T1	In vitro	shATG5	ATP6V1E1	Promoting migration and in vivo metastasis	[20]
Cervical cancer	HeLa	In vivo and In vitro	Serum starvation	SKP1 phosphorylation	——	[117]
Cholangiocarcinoma	RBE, HCCC-9810,SK-CHA-1 and MZ-CHA-1	In vivo and In vitro	shPTEN	TFEB phosphorylation	Cell proliferation and invasion enhancing	[116]
Esophageal squamous cell carcinoma	TE-1 and ECA-109	In vitro	Sulforaphane	mTOR/TFE3	Senescence	[120]
Pancreatic cancer	AsPC-1 and PANC-1	In vitro	GIPC	ABCG2	Resistance to gemcitabine	[115]
Head and neck squamous cell carcinoma	UM-SCC-1, OSC19, HN5	In vivo and In vitro	shBeclin-1/shATG7	——	Inhibiting IL-6 and IL-8 secretion	[109]

### Colorectal cancer

Colorectal cancer (CRC) is one of the most prevalent malignant tumors of the digestive system globally. According to statistical data from 2020, CRC accounted for nearly 1,000,000 deaths worldwide [125]. The development and progression of CRC are associated with the loss of function in tumor suppressor genes and the overactivation of oncogenes [126, 127]. Drug resistance and immune evasion present significant challenges to clinical treatment. In the search for novel therapeutic strategies, researchers have evaluated the anticancer potential of existing drugs and found that thioridazine (THD), an antipsychotic drug, demonstrates strong antitumor activity by inducing apoptosis and inhibiting angiogenesis. Moreover, THD has antimicrobial properties [128–132]. THD-induced autophagy can be reversed in CRC cells by treatment with 3-MA, which reduces LC3^+^ autophagosome formation. Conversely, BafA1 did not significantly alter LC3 expression, indicating that THD-induced autophagy occurs during the early stages of the process without excessive LC3^+^ autophagosome accumulation. This suggests that LC3^+^ autophagosomes are released through secretory autophagy during THD treatment [119]. Furthermore, THD has been shown to induce the secretion of EVs in a time-dependent manner, enhancing oxaliplatin-induced immunogenic cell death [119]. These findings highlight the importance of considering drug dosage and timing in clinical applications. Adjusting the dose and timing to avoid significantly affecting basal secretion could provide further therapeutic options for CRC treatment.

Exosomes are specialized EVs that play a key role in maintaining cellular homeostasis. They mediate functional regulatory processes by targeting adjacent and distant cells, making them crucial indicators for the diagnosis and treatment of CRC [133–135]. Loss of ATG16L1 in HCT-116 cells has been shown to significantly reduce exosome release [20]. Previous studies have demonstrated that ATG16L1 localizes to endosomes, phagosomes, and lysosome-like granules [136]. Its widespread membrane distribution suggests an association with multivesicular bodies (MVBs) independent of double-membrane autophagosomes. In addition to directly regulating ATG16L1, emerging evidence indicates that growth factors and metabolic cues can induce ATG16L1 phosphorylation [137–139], which may provide a theoretical foundation for novel drug development. Molecules targeting ATG16L1 are currently under investigation. For instance, the 3’ untranslated region of ATG16L1 mRNA contains functional binding sites for miR-223. miR-223 mimics can effectively interfere with protein translation *via* post-transcriptional regulation [140]. Similarly, miR-106b and miR-93 have demonstrated comparable functions in regulating ATG16L1 expression [141]. ATG16L1 also shows RAB-binding protein properties, inducing active RAB conformations even in the absence of nucleotide exchange. ATG16L1 and RAB33B mutually influence their membrane localization within organelles [142]. Cargo transport *via* secretory autophagy is ATG16L1-dependent, and miRNA mimics or RAB33B antibodies offer practical and clinically feasible treatment strategies. Furthermore, recent studies have highlighted the role of ATG16L1 in promoting the secretion of exosomal a-disintegrin-and-metalloprotease-10 (ADAM10), contributing to cell protection during bacterial infections. These findings highlight the critical role of ATG16L1 in secretory autophagy [143]. However, whether ATG16L1 is the primary factor driving secretory autophagy in CRC cells remains unclear. Thus, further exploration of the relationship between intestinal microorganisms and secretory autophagy in CRC cells, as well as the potential of targeted therapies, is essential.

### Glioblastoma (GB)

GB is a highly malignant tumor of the nervous system, characterized by a high mortality rate and limited effective treatment options. Following diagnosis, the median survival time is typically less than 15 months [144, 145]. Therapeutic interventions often fail to provide long-term benefits for most patients undergoing extended clinical treatment. Previous studies have highlighted the role of secretory autophagy in cancer therapy, with a specific focus on exosomal substances secreted *via* autophagy. Interestingly, an autophagy-dependent protein secretion process has been identified in glioma cells. Beyond its role in cellular death intervention, autophagy induced by DT-EGF has been shown to regulate necrotic cell characteristics by selectively promoting the release of HMGB1 protein [146]. Interestingly, HMGB1-related secretory autophagy is more readily observed in glioblastomas than in epithelial tumor cell lines, underscoring the need for further studies to elucidate the differences among cell types and the specific mechanisms involved. The release of HMGB1 enhances the immunogenicity of apoptotic cells [147], and when HMGB1 is secreted by dying tumor cells, it augments tumor-specific immune responses through toll-like receptor 4 (TLR4) signaling [148]. These findings suggest that enhancing cellular immunogenicity by modulating specific death mechanisms, such as HMGB1 release, could significantly improve the long-term efficacy of anticancer therapies. Understanding and targeting the mechanisms of secretory autophagy may pave the way for novel therapeutic strategies to overcome this resistance and improve patient outcomes.

Temozolomide (TMZ)-induced autophagic cell death represents a primary mechanism underlying its pharmacological action. In vivo studies have demonstrated that chloroquine (CQ) and HCQ disrupt lysosomal acidification and exhibit anticancer activity in mice. However, combining TMZ with autophagy inhibitors did not significantly improve the overall survival of GB patients [149]. The presence of secretory autophagy redirects cargo from the degradative autophagy pathway to the secretory mode, enabling extensive biological functions. Studies have shown that extracellular HMGB1 protein, released *via* secretory autophagy, acts as a key regulator of crosstalk between tumor cells and the immune microenvironment. TMZ-induced HMGB1 secretion facilitates M1-like polarization of tumor-associated macrophages (TAMs) through the receptor for advanced glycation end-products (RAGE)/extracellular signal-regulated kinase (ERK)/inhibitor of kappa B (IκB)/nuclear factor kappa B (NF-κB) signaling pathway. This polarization promotes the secretion of pro-inflammatory cytokines, such as interleukin-6 (IL-6), interleukin-8 (IL-8), and C-C motif chemokine ligand 2 (CCL2), thereby activating effective anti-tumor immunity [118]. As a cell surface receptor, RAGE is widely expressed on both tumor and immune cells and recognizes multiple secretory substrates, including HMGB1 and S100 proteins, triggering downstream inflammation-related signaling cascades [150–152]. Intracellular HMGB1 is predominantly associated with promoting tumor cell survival, invasion, and drug resistance [153–155]. This dual role of HMGB1 underscores its complex and diverse functions within the TME. Tumor cell-released HMGB1 serves as an immune regulator through classical RAGE-mediated signaling. However, the distinct functional differences between extracellular and intracellular HMGB1 suggest that targeting HMGB1 may not consistently achieve the desired therapeutic effects across different stages of tumor development. Understanding the clear roles of HMGB1 in the TME is critical for developing effective and targeted therapeutic interventions.

### Prostate cancer

Prostate cancer remains the most prevalent solid tumor in men globally and a leading cause of male-specific mortality despite advances in various treatment strategies [156]. Early diagnostic and targeted therapeutic strategies are actively being explored. Serum caveolin-1 (CAV1) levels have been shown to correlate with tumor stage, grade, angiogenesis, and poor prognosis in prostate cancer patients [157–161]. However, the precise mechanisms underlying abnormal CAV1 expression in serum remain insufficiently understood. Recent findings suggest that prostate cancer cells secrete CAV1 through distinct mechanisms. In PC3 cells, CAV1 secretion is dependent on MVBs and plasma membrane fusion mediated by calcium (Ca²⁺) flux, following the classical exosome secretion pathway. On the contrary, in LNCaP cells, CAV1 is secreted *via* both the conventional exosome secretion pathway and an autophagy-dependent mechanism [114]. Exosomes derived from these pathways exhibit heterogeneity, with varying CAV1 expression and distribution patterns depending on the secretion mechanism [162]. Importantly, Ca²⁺ dependency is observed in both cell types, indicating its pivotal role in facilitating vesicle transport [162]. Increased cytosolic Ca²⁺ triggers rapid exosome secretion *via* RAB27a and Munc13-4 [163, 164], with Ca²⁺ influx being influenced by plasma membrane perforation or overexpression of Ca²⁺ transporters. This influx initiates repair cascades involving lysosomal mobilization and fusion with the plasma membrane [162, 165]. Therefore, Ca²⁺ serves as a selective cofactor in exosome release, highlighting the requirement of ion signaling and dynein in the CAV1 transport pathway. The knockdown of ATGs, such as ATG5, ATG9, and ATG12, and the use of autophagosome inhibitors effectively reduce CAV1 secretion in LNCaP cells but not in PC3 cells [162]. For patients with elevated serum CAV1 levels, gene profiling to evaluate ATG expression may provide actionable insights. Patients with high basal autophagy activity could benefit from treatment with 3-MA alone or in combination with calcium channel antagonists to enhance therapeutic efficacy. Similarly, the distribution and localization of CAV1^+^ exosomes could guide the selection of autophagosome inhibitors. Moreover, targeting S100 exosomes with monoclonal antibodies against CAV1 may offer improved outcomes, as demonstrated in preliminary breast cancer studies [166].

The secretory autophagy process of CAV1 may also intersect significantly with glycolysis. As a membrane protein, CAV1 provides a docking site for glycolytic enzymes, including phosphofructokinase (PFK) and aldolase, through its scaffold domain [167]. In vascular smooth muscle cells and lymphocytes, CAV1 cross-links with these enzymes to regulate glycolysis. Metabolomic analyses revealed that intermediate glycolytic metabolites, such as 3-phosphoglycerate, fructose-6-phosphate, and glucose-6-phosphate, were reduced following CAV1 knockdown, indicating their regulatory role in glycolysis [168]. These findings suggest a potential link between CAV1-mediated secretory autophagy and glucose metabolism, although further investigation in prostate cancer is warranted to confirm this association.

### Breast cancer

Breast cancer remains one of the most prevalent solid tumors in women. Despite advancements in medical treatment and the development of molecular-targeted therapy strategies, many breast cancer patients fail to achieve long-term benefits, highlighting the need for identifying new therapeutic targets [169]. Research on exosomes has provided new ways for the diagnosis and treatment of breast cancer. Exosomes are nanoscale vesicles with a bilayer lipid membrane structure, initially thought to serve as a cellular waste disposal mechanism. However, recent studies highlight their key roles in intercellular communication and potential applications in clinical treatment [170–172]. In breast cancer studies, loss of ATG5 was found to significantly reduce exosome release without affecting the cellular uptake of exosomes. This process, intriguingly, was not regulated by ATG7 expression. Mechanistically, ATG5 plays a critical role in recruiting ATP6V1E1 to modulate the acidification of MVBs, thereby controlling exosome production. These ATG5-dependent exosomes significantly enhance the migration and metastasis of breast cancer cells [20]. ATG5, as a component of the E3 ligase, facilitates ATG8 lipidation, a central process in classical autophagy. ATG8 is associated with double-membrane structures (autophagosomes) and single-membrane intracellular vesicles, elucidating the potential roles of ATG5 and ATG8 in maintaining membrane stability and facilitating material transport in non-autophagic pathways [173–176]. Existing literature highlights unresolved questions regarding the influence of ATGs on exosome homeostasis. For example, ATG6 (Beclin1) has been implicated in exosome release during viral infections [22]. ATG6 impacts exosome production by forming complexes with PI3K, promoting ATG14L1 complex formation, and partitioning cargos during MVB formation [177]. This regulatory role is achieved by inducing PI3K association with MVB budding [178]. However, the specific mechanisms underlying the functions of most ATGs in exosome production remain unclear. While ATG5-associated exosome production in breast cancer cells appears to be independent of ATG7, inhibitors of the V1V0-ATPase complex were shown to increase exosome production, even in ATG5-deficient (ATG5^−/−^) cells [179, 180]. This indicates that ATG5-dependent secretory autophagy operates independently of classical autophagy. Importantly, LC3-II expression is not regulated by ATG5, indicating that ATG5 can transport LC3 into exosomes [20]. This observation aligns with the LDELS process described previously. Molecules that directly bind to LC3, ATG5, or other related proteins may undergo selective sorting into MVBs and subsequent release *via* exosomes. Although direct evidence is currently lacking, the role of ATGs in the selective cargo-loading process of exosomes warrants further investigation. Similar to the previously discussed role of CAV1, ATG-related secretory molecules could potentially serve as serodiagnostic markers in breast cancer. This possibility underscores the importance of future studies to elucidate their diagnostic and prognostic utility. It is also noteworthy that the p53 status influences ATG5-related autophagy to some extent [181]. The rational use of arsenic trioxide has been proposed as a potential intervention strategy in this context [182, 183].

In addition to the autophagy-exosome regulatory network, IL-6 secretion is modulated by autophagy in breast cancer. The breast cancer stem cell (CSC) population plays a critical role in promoting malignant proliferation, metastasis, and recurrence [184, 185]. Studies have shown that the activation of ATG7-related autophagy in CSCs facilitates the release of IL-6 [186]. There is a significant positive correlation between IL-6 and pSTAT3 levels in primary breast tumors, as IL-6 triggers the activation of STAT3 signaling [187]. This indicates that autophagy-dependent IL-6 secretion effectively promotes the malignant progression of breast cancer through the STAT3 pathway. However, other IL-6 secretion mechanisms should also be considered, as it remains uncertain whether blocking the autophagy-dependent secretion pathway alone can fully prevent IL-6 secretion. Interestingly, pyrazolopyrimidine sulfamate compounds have been identified as potent and selective inhibitors of ATG7 [188]. These inhibitors provide a promising basis for combination strategies in clinical treatment, potentially offering a new therapeutic avenue for targeting autophagy and its related secretory pathways in breast cancer.

### Cervical cancer

**Fig. 5:**
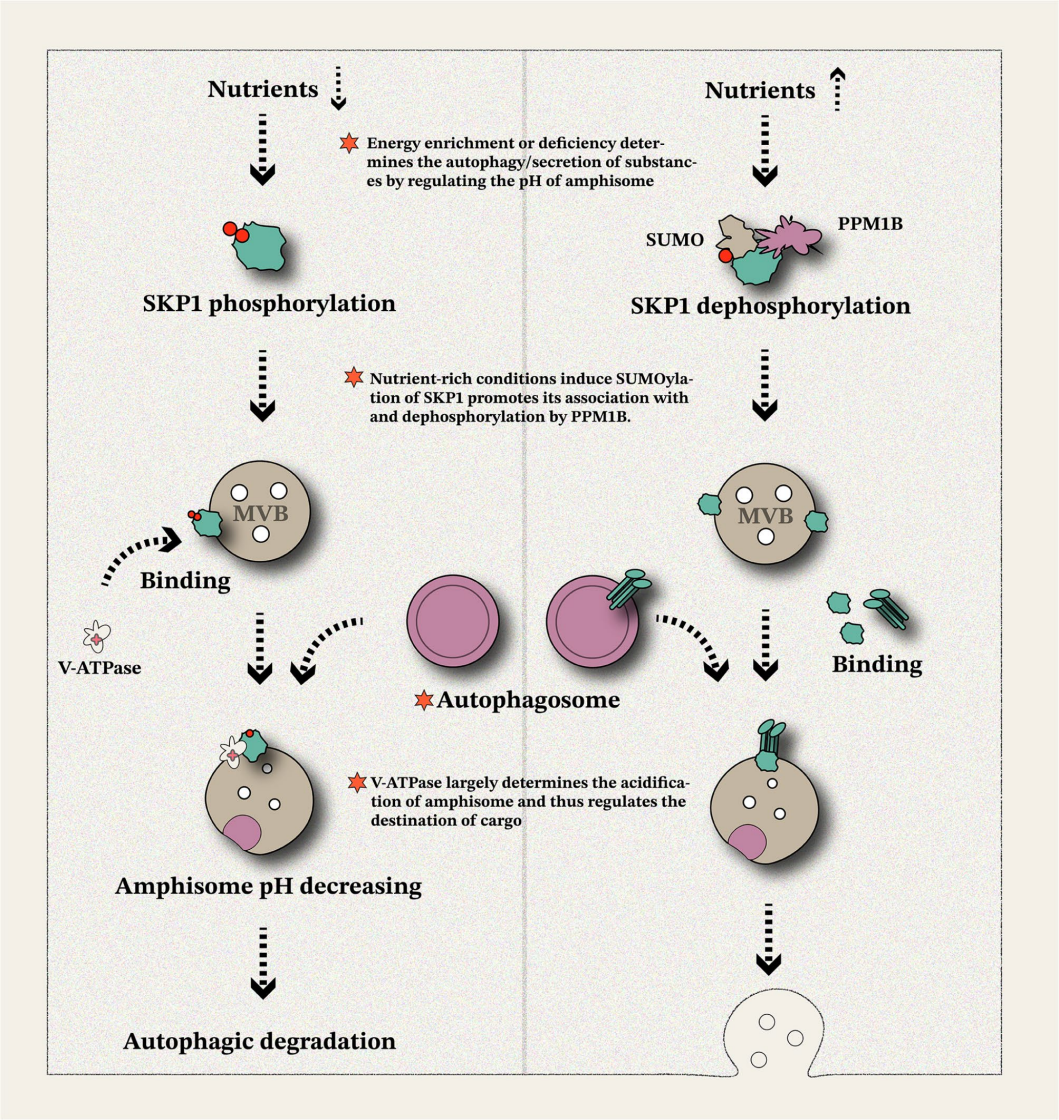
Role of cellular nutrients metabolism in regulating secretory autophagy

Cervical cancer is one of the most significant malignancies affecting women’s health globally. According to previous statistics, over half a million new cases are diagnosed annually, with approximately 90% occurring in low- and middle-income countries [189]. In pursuing targeted therapeutic strategies, researchers have identified several molecular targets and signaling pathways that offer insights for developing conversion therapies [190, 191]. Among these, mTOR, a highly conserved serine/threonine protein kinase, plays a pivotal role as a master regulator of autophagy in multiple cellular processes [192, 193]. In cervical cancer, the activation of mTORC1 signaling effectively inhibits exosomal release, whereas the administration of rapamycin significantly stimulates exosome secretion [194]. Similarly, conditions such as serum starvation and amino acid pretreatment also trigger substantial exosome release [194]. None of these autophagy-inducing factors altered the cargo content of exosomes, indicating that mTORC1’s impact on exosomes in stress-related autophagy models is associated with secretion processes rather than selective cargo loading. The connection between ubiquitinated proteins and secretory autophagy has been elucidated in a separate study. S-phase kinase-associated protein 1 (SKP1), a core component of the SKP1-Cullin 1-F-box protein (SCF) complex, functions as a conserved E3 ligase responsible for the polyubiquitination and proteasomal degradation of numerous proteins [195, 196]. In HeLa cells, SKP1 phosphorylation at Thr131 facilitates its direct binding to V-ATPase, independent of F-box proteins, leading to V-ATPase reorganization and MVB acidification under nutrient-replete conditions [117]. This ultimately triggers the endolysosomal degradation of cargo. Conversely, under nutrient-depleted conditions, SKP1 dephosphorylation occurs, activating a secretory autophagy pathway mediated by SKP1-SEC22B binding within the amphisome compartment [117]. These findings highlight the role of SKP1 in cellular nutrient sensing and its function as a bridge between cellular logistics and metabolism. SKP1 phosphorylation ensures efficient protein degradation and amino acid recycling during energy deficiency, maintaining biosynthetic functions in nutrient-poor environments [197]. On the contrary, reduced SKP1 phosphorylation under sufficient energy conditions promotes excessive transport of metabolic substances *via* autophagy-dependent secretion (Fig. 5). Post-translational modifications of SKP1 appear to coordinate protein breakdown and efflux, enabling rapid cellular stress responses. This regulatory mechanism offers potential strategies for cancer treatment in pathological environments. Furthermore, recent studies have revealed the involvement of ATG5 in secretory autophagy. In HeLa cells, ATG5 recruits ESCRTs to repair damaged lysosomes. Mutations or reductions in ATG5 impair the autophagy-lysosomal pathway, triggering secretory autophagy [198]. Similarly, in ovarian cancer cells, MVB acidification and lysosomal structure/function regulators play critical roles in secretory autophagy. Along with understanding the expression of key molecules, elucidating cellular metabolic mechanisms is vital for translating these findings into clinical applications.

### Hepatocellular carcinoma (HCC)

HCC, primarily caused by chronic liver diseases such as hepatitis B virus infection, is the predominant type of liver cancer. The 5-year survival rate for HCC remains below 50%, with some studies indicating rates as low as 30% for advanced stages [199]. These statistics underscore the urgent need for the development of novel therapeutic strategies. During the exploration of molecular targets, the critical role of ATG family proteins in the initiation and progression of HCC has been increasingly recognized. Several ATGs have been identified as playing pivotal roles in disease development and progression [200–202]. In ATG5 wild-type Huh7 cells, the formation of the ATG5-ATG12 complex enables ALIX to bind with ESCRTs, facilitating lysosomal repair. Upon ATG5 knockout, the ATG3-ATG12 complex adsorbs ALIX, thereby preventing ALIX-mediated lysosomal repair [203]. These findings align with previous reports demonstrating the role of the ALIX-ESCRT system in maintaining lysosomal stability and repairing damaged lysosomes [204–206]. This evidence suggests that the lysosomal damage repair mechanism in HCC cells represents a critical pathway for regulating secretory autophagy, particularly through lysosomal damage repair and quality control. In addition to the classical role of V-ATPase in regulating intracellular pH and influencing material transport outcomes, other lysosome-associated proteins contribute to similar regulatory functions. For example, lactate dehydrogenase B (LDHB) is highly expressed in cancer cells and catalyzes lactate metabolism to control lysosomal acidification and vesicle maturation [207]. Although this mechanism is observed in both oxidative and glycolytic cancer cells, its applicability may be limited. Hypermetabolic cancer cells are likely to depend more heavily on this metabolic coupling mechanism for survival and progression. These insights into the role of lysosomal repair and metabolic coupling in HCC highlight potential therapeutic targets for addressing the challenges posed by this aggressive disease.

### Cholangiocarcinoma (CCA)

CCA is a highly heterogeneous tumor of the digestive system. Due to the high heterogeneity of tumor cells and significant drug resistance, the prognosis and survival time of CCA patients are severely compromised. The incidence of CCA has remained persistently high over the past decades [208, 209]. As a refractory tumor, current studies focus on innovative strategies, including targeted therapies for protein degradation pathways [116, 210, 211]. The proteasomal degradation pathway and the autolysosome-related degradation pathway represent two parallel yet interconnected systems for protein degradation in eukaryotic cells. The tumor suppressor phosphatase and tensin homolog (PTEN) has been shown to inhibit the ubiquitin-mediated proteolytic system while promoting the lysosomal degradation pathway in CCA cells [116]. PTEN primarily relies on its lipid phosphatase activity to modulate TFEB^Ser211^ phosphorylation, independent of PI3K/AKT signaling, thereby enhancing lysosome biogenesis and acidification. When PTEN is deficient, the lysosomal degradation of MVB cargos is impaired, leading to the production of exosomes. These functional exosomes further facilitate cell proliferation and invasion [116]. The transcription factor EB (TFEB) has a conformation that enables direct binding to the coordinated lysosomal expression and regulation (CLEAR) element, promoting the expression of genes associated with lysosomal function. TFEB activation or overexpression significantly enhances lysosome-related protein expression, thereby increasing lysosomal activity [212, 213]. Phosphorylation of TFEB serves as a critical regulatory mechanism for its cellular localization and activity. Two serine residues, Ser142 and Ser211, determine TFEB’s state. When both residues are phosphorylated, TFEB remains inactive and localized in the cytoplasm. Dephosphorylation of either residue activates TFEB, allowing it to enter the nucleus to regulate transcription [214–216]. These findings highlight the importance of post-transcriptional modifications in secretory autophagy. High CD63 expression and low PTEN levels in CCA tissues are associated with poor patient prognosis, indicating a relationship between PTEN and exosome production during secretory autophagy. PTEN is essential for regulating lysosomal function, suggesting that lysosome acidification regulators are key mediators restraining MVB-lysosome fusion and exosome release [116]. PTEN loss or overexpression has been identified as a prognostic marker for evaluating CCA responsiveness to proteasome inhibitors [217]. Although PTEN-mediated secretory autophagy in CCA is independent of AKT signaling, the cholangitis-to-CCA continuum requires both AKT signaling and Aurora kinase A (Aurka) [218]. Pharmacological inhibition of Aurka effectively halts disease progression [218]. This study highlights the multi-faceted regulatory role of PTEN in CCA progression and underscores its significance in the interplay between tumor cells and their microenvironment.

### Esophageal squamous cell carcinoma (ESCC)

Esophageal cancer is categorized into two pathological types: ESCC and adenocarcinoma. ESCC primarily develops from precancerous lesions, with a 5-year survival rate of less than 20%, highlighting the need for molecular-targeted therapies [219]. Developing novel compounds targeting cell death pathways offers a promising therapeutic strategy for the clinical management of ESCC. Cellular senescence, a mechanism of cell death, prevents tumorigenesis and progression by inhibiting cell proliferation, making senescence-inducing agents a promising anti-cancer strategy, particularly in combination with pro-senescence drugs and conventional therapies [220]. Sulforaphane (SFN), an isothiocyanate derived from cruciferous vegetables, shows significant anti-tumor activity. Mechanistically, SFN modulates the mTOR/TFE3 axis to inhibit autophagy by regulating lysosome biogenesis and triggering the secretion of functional exosomes, which induce senescence in surrounding cells through paracrine signaling [120]. Transcription factor binding to IGHM enhancer 3 (TFE3), a master regulator of lysosomal activity, is regulated by mTORC1-dependent multisite phosphorylation (TFE3^Ser321^) [221]. SFN activates mTOR signaling, inducing TFE3 phosphorylation, which is crucial for initiating secretory autophagy. This underscores the critical role of mTOR signaling in secretory autophagy. SFN’s role in anti-tumor therapy has become increasingly evident, including its ability to improve inflammatory responses and inhibit pseudopodia formation in tumor cells [222, 223]. Combining SFN with various clinical chemoradiotherapy strategies could provide new therapeutic approaches. However, the emergence of senescence-associated secretory phenotype (SASP), which triggers the release of inflammatory factors, may partially counteract SFN’s anti-tumor effects [224]. Careful consideration of SASP-related impacts is essential when designing SFN-based therapeutic strategies.

### Pancreatic cancer

The incidence of pancreatic cancer doubled between 1990 and 2017, with the risk increasing with age and being higher in high-income countries [225]. Effective treatment options for pancreatic cancer remain limited, particularly for advanced-stage patients. Targeted therapy has emerged as a promising approach, with GAIP-interacting protein C-terminus (GIPC) identified as a potential candidate target. GIPC functions as a scaffold protein through its PDZ motif, regulating receptor-mediated transport. Upon receptor internalization, GIPC directly participates in vesicle trafficking by interacting with endocytic vesicles near the plasma membrane [226]. A study has shown that GIPC is involved in the autophagy-exosome interaction within pancreatic cancer cells. Although Beclin1 and ATG7 expression levels remain unchanged in GIPC-deficient cells, these cells exhibit an excessive accumulation of MVBs, leading to increased exosome secretion [115]. Exosomes derived from GIPC-deficient cells contain ATP-binding cassette transporter G2 (ABCG2), a molecule associated with resistance to gemcitabine treatment [115]. ABCG2, a member of the ABC transporter superfamily, is overexpressed in many tumors and plays a critical role in the efflux of various chemotherapy drugs, contributing to the multidrug resistance (MDR) phenotype [227, 228]. Understanding the relationship between GIPC and ABCG2 in secretory autophagy requires immunohistochemical analysis in clinical samples. Such analysis could elucidate their expression and distribution patterns, aiding the development of more effective targeted therapy strategies. However, the heterogeneity of exosomes must be considered, as alterations in ABCG2 expression may be limited to GIPC-deficient cells. Single EV sequencing provides a potential solution to this challenge, offering higher resolution to analyze exosome characteristics [229–231].

Stromal fibrosis disrupts vasculature, resulting in intratumoral hypoxia and metabolic imbalance. Tumor cells must adapt their nutrient acquisition and utilization strategies to sustain rapid proliferation. Studies have demonstrated that pancreatic stellate cells (PSCs) release significant quantities of alanine to support pancreatic ductal adenocarcinoma (PDAC) cells *via* secretory autophagy [232]. Autophagy activation in PSCs is induced by stimulation from PDAC cells, triggering PSCs to catabolize proteins and produce alanine, which is then released into the TME through a secretory autophagy pathway. When shATG5 and shATG7 were used to inhibit autophagy, alanine secretion was significantly reduced [232]. PDAC cells actively uptake alanine and utilize it as a carbon source in the tricarboxylic acid (TCA) cycle, converting it into pyruvate. Alanine-derived pyruvate does not equilibrate with cytosolic pyruvate, providing a direct carbon source for mitochondrial metabolism without affecting the intracellular NAD^+/^NADH redox balance [232]. This metabolic reprogramming enables the efficient diversion of conventional carbon sources, such as glucose, to other biosynthetic pathways, including serine and glycine production, which are crucial for nucleic acid synthesis [232]. This synergistic metabolic adaptation provides essential substrates for tumor growth in nutrient-restricted environments. In highly mesenchymal PDAC, the reciprocal interaction between stromal cells and tumor cells improves the energy acquisition mechanisms of tumor cells under malnourished conditions. This “predatory demand” highlights a survival strategy for tumor cells within extreme microenvironmental constraints.

### Head and neck squamous cell carcinoma (HNSCC)

HNSCCs are solid malignancies originating from the oral cavity, pharynx, and larynx. Their etiology is often associated with excessive exposure to carcinogens and alcohol consumption [233]. The interaction between cancer cells and their microenvironment plays a critical role in tumor survival and malignancy. Over the past decade, the secretory properties of cancer-associated fibroblasts (CAFs) have posed significant challenges in the treatment of tumors. Studies have demonstrated distinct differences between the characteristics of CAFs and normal fibroblasts (NFs) within the same anatomical site. CAFs show a higher abundance of concentrated vesicle structures, indicative of robust secretory activity [109]. Knockdown of Beclin-1, ATG7, or treatment with CQ has been shown to significantly reduce the secretion of cytokines such as IL-6 and IL-8 [109]. These cytokines are systemically elevated in HNSCC patients and are correlated with resistance to targeted therapies. Moreover, IL-6 and IL-8 are secreted by stromal fibroblasts in various cancer types and are implicated in senescence-related secretory phenotypes [234–237]. Despite the high autophagic activity observed in primary CAF cells, inhibition of autophagy through Beclin-1 knockdown effectively reduces secretory autophagy, thus lowering IL-6 and IL-8 levels [109]. Evidence from extensive literature has demonstrated that both chemotherapy and radiotherapy are potent inducers of autophagy in HNSCC cells [238, 239]. While autophagy may act as a protective mechanism for tumor cells, secretory autophagy contributes to clinical resistance to chemotherapy and radiotherapy. However, clinical trials using autophagy inhibitors such as CQ have reported limited efficacy due to insufficient intratumoral concentrations to effectively inhibit autophagy within the tumor microenvironment [240]. To address these limitations, the development of novel small-molecule drugs has become a crucial focus. SAR405, a PI3K class III inhibitor, has demonstrated potent autophagy inhibition in HNSCC and CAFs at low doses (1.0 μM), effectively limiting autophagy levels [109]. This compound represents a promising alternative to CQ and may offer a viable therapeutic strategy in the clinical setting to overcome resistance associated with secretory autophagy.

## Secretory autophagy and tumour microenvironment stress

### Hypoxia

Tumor cell proliferation requires sufficient oxygen supply, and cells located more than 100 μm from an oxygen source are considered hypoxic [241]. Hypoxia is one of the most common pathological features of solid tumors, contributing to tumor invasion, metastasis, and resistance to therapy [242–244]. CAFs, key components of the TME, facilitate cancer progression through various mechanisms, particularly *via* their secretome. Secreted factors such as stromal cell-derived factor 1 (SDF-1), interleukin-32 (IL32), and transforming growth factor-beta (TGF-β) are critical mediators of intercellular communication within the TME [245, 246]. Recent studies have demonstrated that ataxia telangiectasia-mutated (ATM) protein is phosphorylated under hypoxic conditions, leading to autophagosome accumulation and lysosomal dysfunction. This process facilitates the fusion of autophagosomes with MVBs through ATG6V1G1, promoting the release of autophagy-dependent exosomes [247]. Phosphorylated ATM also induces the phosphorylation of BNIP3, forming the BNIP3-P300-FOXO3 complex, which transcriptionally upregulates ATG5 and ATG16L expression [247]. These findings suggest that hypoxia-induced ATM phosphorylation drives secretory autophagy through two distinct mechanisms: enhancing autophagosome formation and modulating MVB acidification. MVB acidification, a critical step in secretory autophagy, is closely associated with the function of V-ATPase. Hypoxic CAFs further promote the secretion of G-protein coupled receptor 64 (GPR64)-enriched exosomes *via* the secretory autophagy pathway, enhancing cancer cell invasion through NF-κB signaling [247]. Although the exosomal loading process of GPR64 remains unclear, it is hypothesized that GPR64, as an adhesion receptor [248], may be sorted by interactions with other membrane proteins. These findings highlight the importance of hypoxia-driven secretory autophagy in tumor progression and highlight potential therapeutic targets within this pathway.

### Mechanical stress

The mechanisms underlying tumor metastasis and colonization are complex, with only approximately 0.01% of circulating tumor cells successfully forming secondary lesions due to the challenges of overcoming fluid shear stress [249, 250]. Tumor cells experience acute shear stress (ASS) during growth and metastasis, whether *via* blood or lymphatic dissemination. Under normal physiological conditions, tumor cells exhibit basal levels of autophagy and exosome release. However, changes in the mechanical microenvironment, such as ASS, trigger the remodeling of autophagy-dependent degradation processes, thereby associating autophagy with EV release. Studies have demonstrated that ASS promotes the fusion of autophagosomes with MVBs, resulting in the calcium-dependent exosomal release of LC3II^+^ and LAMP1^+^ exosomes [251]. The presence of autophagy-related proteins in exosomes, including p62, LC3, and LAMP2, highlights the close relationship between autophagy and exosomal pathways [252–256]. Interestingly, the cellular response to mechanical signals is mediated by calcium transporters such as PIEZO1, which helps maintain cellular homeostasis under stress [257–259]. The balance between autophagosomes and MVBs is essential in secretory autophagy, as it determines the fate of transported substances. Blocking exosome release under shear stress conditions leads to the accumulation of autophagosomes and MVBs, ultimately activating Caspase3/PARP-mediated apoptosis [251]. These findings suggest that exosomes serve as a complementary pathway to maintain cellular homeostasis, particularly when autophagy is insufficient to degrade large quantities of damaged proteins and prevent cell death. The regulation of exosome release by ASS highlights how tumor cells adapt to mechanical stress and contribute to the formation of a metastatic microenvironment. These insights emphasize the interplay between autophagy, exosomal release, and tumor cell survival under mechanically stressful conditions, providing potential targets for therapeutic intervention.

## Secretory autophagy and immune regulation

**Fig. 6:**
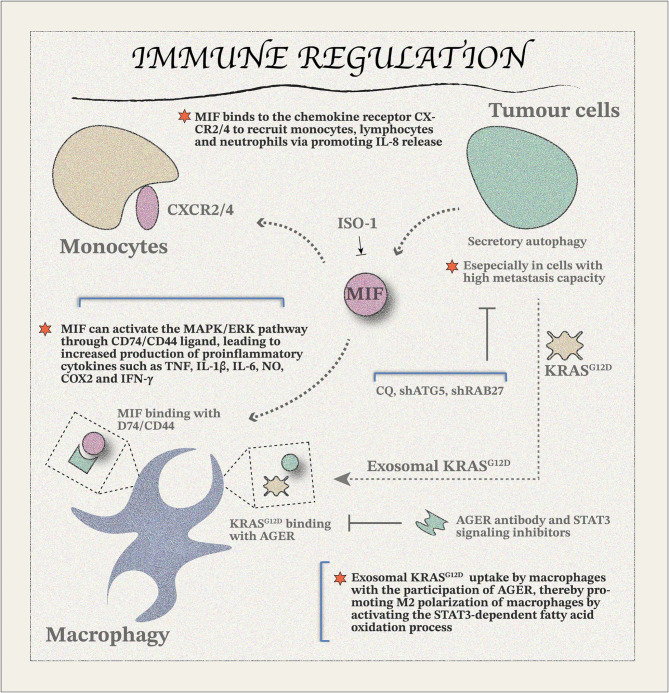
Immune regulation of secretory autophagy molecules from tumour cells

The emergence of tumor cells triggers immune system activation, leading to immune recognition and subsequent elimination through immune-mediated killing processes. However, malignant cells accumulate oncogenic mutations to escape immune surveillance, downregulate tumor antigens, upregulate pro-survival genes, and reshape the immune microenvironment, enabling evasion of immune detection and elimination [260–262]. Autophagy inhibitors are currently among the key strategies for overcoming resistance to clinical treatments. However, they also contribute to immune tolerance in certain contexts. Exosomes have been identified as crucial mediators in enhancing the tumor immunosuppressive microenvironment in this complex landscape. Tumor-derived exosomes can create a tolerant microenvironment, facilitating interactions with the immune system [263, 264]. Understanding the malignant potential of tumor cells and their interplay with exosomes is critical for advancing therapeutic strategies. Studies have shown that tumor cells with high metastatic capacity show elevated basal autophagy levels accompanied by increased secretion activity (Fig. 6). Inhibition of autophagy results in the accumulation of damaged mitochondria and increased reactive oxygen species (ROS), which then enhance secretory autophagy of macrophage migration inhibitory factor (MIF) [265, 266]. MIF is a key immune regulatory molecule with essential roles in both innate and adaptive immunity [267, 268]. It is secreted by various cells, including epithelial and endothelial cells [268]. Interestingly, MIF lacks an ER localization motif in its structure and is, therefore, not secreted through the classical ER/Golgi pathway [269]. Instead, MIF is secreted *via* an unconventional protein secretion pathway, which has been implicated in cancer progression [270, 271]. MIF promotes target cell signaling through multiple receptors. For instance, MIF activates the MAPK/ERK pathway *via* the CD74/CD44 ligand, resulting in the increased production of proinflammatory cytokines such as TNF, IL-1β, IL-6, nitric oxide (NO), cyclooxygenase-2 (COX2), and IFN-γ, thereby triggering immune responses through autocrine and paracrine signaling [268]. Furthermore, MIF binds to chemokine receptors CXCR2 and CXCR4, recruiting monocytes, lymphocytes, and neutrophils by promoting IL-8 release [268]. In addition to the knockdown of ATG7 and treatment with CQ, the use of antioxidants has been shown to block MIF-induced secretory autophagy. Thus, the combined use of autophagy inhibitors and MIF inhibitors, such as ISO-1, may provide novel insights into enhancing anti-tumor immunity. The effectors released *via* the secretory autophagy pathway significantly contribute to the formation of an immunosuppressive microenvironment by recruiting immune cells and releasing inflammatory factors. This highlights the critical need for therapeutic strategies targeting both secretory autophagy and its downstream effectors to disrupt the tumor-promoting immune landscape and improve clinical outcomes.

*KRAS*, one of the most frequently mutated oncogenes in human solid tumors, plays a critical role in immunomodulation associated with secretory autophagy. Aberrant *KRAS* mutations are prevalent in various solid tumors, including pancreatic, lung, and colorectal cancers [272]. Activation of oncogenic *KRAS* signaling leads to immunosuppression through alterations in the TME. PDAC, primarily driven by *KRAS* mutations, remains a highly lethal malignancy despite advances in clinical trials. The survival rate of PDAC patients has shown minimal improvement, highlighting the need for novel therapeutic approaches. Macrophages, quiescent in their basal state, can differentiate into two subtypes upon external stimulation: the M1 phenotype, which shows anti-tumor properties, and the M2 phenotype, which supports tumor progression [273]. Recent studies have focused on preventing the transformation of macrophages into the tumor-promoting M2 phenotype. In PDAC, the G12D mutation in *KRAS* (*KRAS*^G12D^) is the most common alteration. Emerging evidence indicates that autophagy activation facilitates the release of exosomal *KRAS*^G12D^ from PDAC cells, which, upon uptake by macrophages through advanced glycation end-product receptor (AGER), induces M2 polarization. This process is mediated by activation of STAT3-dependent fatty acid oxidation [274]. AGER plays a pivotal role in the malignant progression of PDAC and its resistance to therapy [275–277]. Depletion of AGER has been shown to limit *KRAS*^G12D^-driven PDAC progression and enhance chemotherapy sensitivity [275–277]. Targeting the AGER-mediated polarization pathway through molecular drug design could serve as a promising approach to inhibiting tumor progression. Preliminary in vitro studies have validated measures such as using AGER antibodies, STAT3 signaling inhibitors, CQ, shATG5 and shRAB27 to block secretory autophagy-related signaling pathways of *KRAS*^G12D^ [274]. The development of innovative strategies targeting the delivery of *KRAS*^G12D^ protein offers a viable avenue for therapeutic intervention. However, exosomes, which play a key role in this process, share mechanistic features with other types of EVs, complicating their precise isolation and identification. Addressing this challenge is essential for advancing the application of exosome-targeted therapies in clinical settings. Further research into purification methods and high-precision identification of exosomes is important to refine and optimize these therapeutic strategies.

## Secretory autophagy and therapy resistance

Secretory autophagy, particularly involving HMGB1, plays a key role in mediating the crosstalk between tumor cells and clinical treatments, affecting tumor sensitivity to therapeutic agents such as TMZ. Extracellular HMGB1, released *via* secretory autophagy, has been identified as a key regulator in this process [118]. Studies have shown that autophagy inhibitors like LY294002 and 3-MA significantly block TMZ-induced autophagosome formation and suppress the autophagy-dependent secretion of HMGB1 [118]. These findings provide a strong rationale for the combination of autophagy inhibitors with TMZ to enhance therapeutic outcomes. Furthermore, the loss of ATG5 or GORASP2 in GB cells significantly reduces HMGB1 secretion during TMZ treatment [118]. ATG5-mediated activation of classical autophagy and GORASP2-dependent unconventional secretion pathways are essential for cytoplasmic protein efflux [51, 278, 279]. This suggests that HMGB1 is located within autophagosomes in GB cells, though the precise mechanism of its loading into autophagosomes warrants further investigation. HMGB1 primarily resides in the nucleus, where it functions as a DNA chaperone. Loss of HMGB1 can lead to excessive DNA damage and reduced DNA repair efficiency during chemotherapy [280]. Targeting ATG5 or inhibiting autophagosome formation may provide a viable therapeutic strategy to improve treatment outcomes. However, such approaches may lack selectivity due to the diverse nature of autophagic cargo. A deeper understanding of the HMGB1 loading process could enhance therapeutic precision and efficacy. ATG7 is another crucial regulator of cargo release during secretory autophagy. Its interaction with ATG5 has been shown to facilitate the translocation of CFTR to MVBs, triggering autophagy-dependent CFTR release *via* the ESCRT pathway [281]. This process requires SEC16 homolog A (SEC16A) (ER export factor) to disengage CFTR from the ER during unconventional autophagy [282]. Furthermore, the coupling of ATG3 and ATG12 is critical for secretory autophagy [283, 284]. The secretion of cytokines such as IL-1β and leukemia inhibitory factor (LIF) is significantly correlated with ATG7 expression, highlighting the interplay between autophagy-dependent secretion and immune modulation [285–288]. IL-1β regulates tumor therapeutic responses and immune activation [289, 290], while the LIF-mediated secretory pathway contributes to drug resistance in tumor cells [291, 292]. These findings suggest an indirect link between autophagy-dependent cargo secretion and tumor resistance to treatment, emphasizing the need for targeted interventions to mitigate drug resistance mechanisms.

## Treatment strategies on secretory autophagy

Targeting secretory autophagy still lack of sufficient clinical evidences to promote its clinical transformation. Therefore, this part mainly proposes new strategies for targeted therapy based on the aforementioned secretory autophagy mechanism of tumour cells, in order to better promote clinical transformation. At present, the approaches to target secretory autophagy are still in explorational stage, and the main way targeting secretory autophagy is still rely on the traditional signal shared with autophagy. For example, the using of inhibitors of PI3KC3/VPS34 [293], targeting ATG4 [294, 295], ATG7 [296], ATG5 [297], and ULK1-ULK2 complex [298], these small molecule drugs have shown good anti-tumour activity and high safety profile. However, these drugs are all based on targets related to classical autophagy signaling and thus lack specificity. In addition, there are drugs that target lysosomal acidification to promote secretory autophagy by blocking the conventional autophagy pathway. For example, targeting ATG5 [297, 299], ATP6V1E [300, 301], ATP6V1G [247, 302], TFEB [116, 303, 304], ATG6V1G [247, 305] could block the fusion of autophagosomes with lysosomes and promote autophagy-dependent secretion. Some classical autophagy inhibitors have also shown good anti-tumour effects and can act on the secretory autophagy pathway. Currently, the only drug that target autophagy in clinical practice is CQ/HCQ. These drugs can inhibit autophagic flux by deacidification of lysosomes and blocking the fusion of autophagosomes and lysosomes. CQ can also enhance the sensitivity of chemotherapy drugs independent of autophagy pathway [306–308]. Clinical trials have found that inhibition of autophagy can improve the prognosis of patients with glioblastoma. Compared with the control group, CQ combined with radiotherapy and the alkylating agent temozolomide could significantly improve the median survival time of patients (33 months vs. 11 months) [309]. Phase I clinical trial results also demonstrated that the combination of HCQ and temsilimus can improve the prognosis of patients with advanced solid tumours and melanoma [310]. Preliminary application in other tumours and combined therapy trials are gradually carried out, and preliminary results have been achieved. These small molecule drugs around the common pathway of classical autophagy and secretory autophagy have shown good therapeutic effects, but they lack specificity. These small molecule drugs around the common pathway of classical autophagy and secretory autophagy have shown good therapeutic effects, but they lack specificity. The targeted research on secretory autophagy is in the exploratory stage, and further research is needed. However, the targeting of secreted autophagy-related cargo has provided new insights. For example, to prevent cargo loading or targeting molecules itself, we launched discussion.

## Strategy 1: targeting releasing agents

### Targeting HMGB1

Salicylates and glycyrrhizic acid are key HMGB1 inhibitors, and it has been widely used in clinical practice, and its safety is worth to be confirmed [311, 312]. The key step is to analyze the correlation between salicylates/glycyrrhizic acid and tumour occurrence and progression in the long-term treatment of tumour. Another study showed that ethyl pyruvate could inhibit the phosphorylation and release of HMGB1, thereby inhibiting the biological function of HMGB1 [313]. Metformin, another drug commonly used in clinical practice, was also found to bind HMGB1 and interfere with its function [314]. Although several drugs have been found to interfere with HMGB1 expression, it should be of concern that the function of HMGB1 is differentiated between intracellular and extracellular.

### Targeting KRAS

KRAS, as one of the key mutated genes in solid tumours, provides a lot of hope for the field of tumour targeted therapy. The mechanism of secretory autophagy regulation of KRAS^G12D^ has been described previously, and new therapeutic approaches targeting KRAS^G12D^ have emerged. TH-Z827 (IC50:4.4 μm) and TH-Z835 (IC50:2.5 μm), as newly developed KRAS^G12D^ inhibitors, have lower pharmacological concentrations and certain safety profiles, and significantly improve the efficacy of immunotherapy in animal experiments in vivo [315]. A noncovalent, potent, and selective KRAS^G12D^ inhibitor, MRTX1133(IC50:5 μm), was also identified in another study [316, 317]. MRTX1133 shows an excellent tumour suppressor effect in pancreatic cancer. MRTX1133 can reprogram the tumour immune microenvironment and enhance the immune killing effect of FAS-mediated CD8^+^T cells [318]. In addition to KRAS^G12D^, hot spot mutations such as G12C also widely exist and play a role in carcinogenesis. Although whether the secretion of KRAS G12C depends on secretory autophagy is not known, its application value has also been clarified in the treatment of KRAS^G12C^. Data from sotorasib, a KRAS^G12C^ inhibitor, in KRAS^G12C^-mutated advanced lung cancer showed that 88.1% (52 patients) of the patients had disease control [319]. These results suggest that KRAS targeted inhibitors may play an anti-cancer/immuno-synergistic role in blocking the secretory autophagy signaling process of KRAS.

### Targeting ADAM10

ADAM10 inhibitors LT4 and MN8 have been found to inhibit cell metabolism and energy production, as well as cell malignancy in Hodgkin’s lymphoma [320]. ADAM10 inhibitors LT4 and CAM29 were also reported in another study, both of which could enhance the anti-tumour immunity of targeted drugs by increasing the expression of CD30 [321]. LT4 and MN8 may have higher biological activity than GI254023X, another commercial drug, which also exerts anti-tumour effect through ADAM10 [322]. However, the safety and pharmacological parameters evaluation data need to be further analyzed.

### Targeting CAV1

At present, targeted drugs/small molecule inhibitors of CAV1 have not yet entered the clinic, and there is a lack of specific drugs to interfere with its function. Studies have found that chrysotobibenzyl can inhibit CAV-1, inhibit the EMT process of lung cancer cells, prevent cell metastasis and promote the efficacy of cisplatin [323]. An interesting phenomenon also exists during the maintenance of cancer stem cells, nitric oxide (NO) is a bioactive substance that abnormally accumulates in tumours and is associated with tumour progression. In CSCs, NO can inhibit the binding of OCT4 to CAV1, thereby inhibiting the degradation of OCT4 and promoting its stemness [324]. Thus, blocking the NO transduction pathway may provide a feasible option for targeting CAV1. Similarly, Triptolide can also regulate the malignant ability of prostate cancer cells through CAV1 [325]. However, the IC50 and specificity of triptolide are still unclear and need to be further improved in the future. Similarly, Simvastatin can inhibit castration-resistant prostate cancer metastasis and enhance the efficacy of androgen receptor antagonist therapy by inhibiting CAV1 expression [326]. The emergence of traditional medicine may provide guidance for the development of small molecule drugs. However, the pharmacological evaluation of different drugs is still lack of research, and the bioavailability and effective pharmacological concentration of drugs are still unclear.

### Targeting MIF

MIF inhibitors have been extensively studied. At present, the most commonly used MIF inhibitors are isoxazolines (ISO-1), which were first discovered in 2002, ISO-1 inhibits MIF function through dose-dependent regulation with an IC50 of about 7 μM [327]. Another study showed that ISO-1 (10 μm) inhibited MIF expression by up to 40%, indicating a large regulatory network [328]. The in vivo study demonstrated that ISO-1 significantly reduced the malignant potential of prostate cancer, colon cancer and melanoma [329–331]. Another ISO-1 derived compound, CPSI-1306, lacks the characteristic phenolic function, thus making the drug less susceptible to bioconjugation reactions and inhibiting its inactivation and excretion in vivo [332]. In the subsequent development process, Alam-4b (IC50:7.3 μm) and ISO-66 (IC50:1.5 μm) have also been gradually found, ISO-66 can significantly inhibit tumour growth with low/no toxicity in melanoma and colon cancer [333, 334]. The above MIF inhibitors are all isoxazoline derivatives, and the development and application of small molecule MIF inhibitors targeting these molecules have the potential to be new therapeutic methods. With the further development of basic research, 1,2,3-triazole derivatives Jorgensen-3 g and Jorgensen-3 h were found to have an IC50 of only 1 μm, based on which Dziedzic-3bb was more biocompatible [335, 336]. However, in vitro and in vivo studies on these small molecules are still lacking.

## Strategy 2: changing the cargo loading

At present, the cargo selectivity of secretory autophagy still needs to be explored, and the targeted therapy for sorting proteins also needs to be explored in depth, which requires multidisciplinary progress and a deep understanding of protein structure. Few studies have revealed modulators of secreted autophagy-related sorting proteins, for example, TRIM16 is a secreted autophagy sorting protein. Although highly effective drugs targeting TRIM16 have not been reported, study have found that a natural compound may affect its function. Withaferin A, a steroid ester extracted from ashwagandha, has been found to act as a TRIM16 regulatory drug to block tumour progression [337]. However, the targeted research of TRIM16 still needs multidisciplinary integration and common development.

## Limitations: thorny issues in secretory autophagy

Secretory autophagy is a critical process in tumor progression. The metabolic remodeling of tumor cells often results in the abnormal accumulation of intermediate metabolites. While some of these substances are broken down and recycled through degradation pathways such as autophagy, a significant proportion is transported outward *via* secretory pathways. These substances, whether actively or passively transported, supply energy and substrates to nutrient-deficient cells and redistribute essential resources. As signaling molecules, they also deliver critical information to nearby and distant cells, enabling tumors to adapt synergistically to various adverse environments. Thus, secretory autophagy plays a key role in maintaining the balance between degradation and secretion within tumors. Disruption of this balance often leads to compensatory changes, such as altered expression or post-transcriptional modifications of key regulatory molecules, to re-establish equilibrium. Several unresolved issues remain regarding secretory autophagy: (1) Tumor cells appear to coordinate autophagy and secretion through the expression or modification of key mediators such as ATG5 and ATG7. However, the precise mechanisms underlying these changes remain unknown. (2) The process of cargo selection suggests the presence of specialized carriers. The mechanisms by which cells select specific substances for secretion and the rationale behind these decisions are not yet fully understood. (3) Under stress, cells typically rely on autophagy to recycle materials and generate energy. Yet, in extreme environments, cells paradoxically secrete large quantities of substances, including glucose metabolism intermediates and various lipids, rather than using them internally [3, 171, 338]. This phenomenon raises questions about the factors driving the choice between self-utilization and secretion. Emerging technologies offer promising tools to address these challenges [339, 340]. Single-cell sequencing provides insights into the heterogeneity of secretory preferences among different cell populations, helping identify which subgroups prioritize secretion [341, 342]. Similarly, organoid technology offers realistic and feasible models for in vitro studies, enabling detailed exploration of the regulatory mechanisms underlying secretory autophagy [343–345]. These advancements could pave the way for a deeper understanding of secretory autophagy and its role in tumor progression, potentially leading to novel therapeutic strategies.

## Can secretory autophagy improve adverse reactions?

Targeting autophagy presents new therapeutic options for clinical cancer treatment. Although low basal autophagy is a metabolic mechanism that helps cells maintain internal homeostasis, tumor cells exploit this process. Adaptive responses triggered by hypoxia and therapeutic stress can lead to excessive autophagy activation, enhancing tumor resistance to treatment. Sustained high autophagy levels in advanced tumors, coupled with the induction or inhibition effects of chemotherapy drugs, suggest that autophagy acts as a key resistance mechanism. Excessive autophagy activation is often the primary means by which cells counteract drug stress. While autophagy inhibitors, such as CQ, have shown potential in preventing autophagy by targeting lysosomes, they are associated with significant side effects. For instance, long-term CQ use has been linked to blurred vision, prolonged QT intervals on ECG, nausea, and abdominal cramps [346]. The secretory autophagy pathway offers an explanation for some of these adverse effects. When autophagy is blocked, secretory activity may increase, releasing large quantities of substances that may promote tumor evolution under drug stress. This adaptation enables tumors to accumulate mutations and refine metabolic characteristics, further complicating treatment. Understanding the regulatory mechanisms of secretory autophagy may help mitigate these side effects and improve clinical outcomes. For example, CQ has been shown to trigger the secretion of autophagy-related protein ATG8 [347], which can be taken up by neighboring cells, potentially initiating autophagy in those cells. Combining CQ with secretion-associated dynein inhibitors could achieve better efficacy [348–350]. Furthermore, more specific drugs with minimal off-target effects need to be developed. Targeted drugs with single pharmacological mechanisms may represent a future direction for treatment strategies. Cell-penetrating peptide (CPP)-based delivery systems offer a promising approach for delivering membrane-impermeable drugs, peptides, proteins, and nucleic acids. CPPs, containing 4–40 amino acids, can modify mRNA to enhance delivery to cells and tissues [351, 352]. Therefore, molecular peptide modification targeting key molecules involved in the secretory autophagy process could improve bioavailability and intracellular delivery, providing valuable insights for future targeted therapies.

## Perspective and future application

The dynamic balance between cancer cells and the TME is a critical driver of cancer progression. Over the past decade, the regulatory mechanisms of secretory autophagy have revealed significant connections between autophagy and secretory pathways. Secretory autophagy facilitates the release of numerous inflammatory factors, which are crucial for tumor development. For instance, excessive secretion of IL-6 and IL-8 has been observed in various cancers and is associated with resistance to targeted therapies and poor prognosis [234, 235, 353–357]. Extensive literature indicates that chemotherapy and radiotherapy can induce cytoprotective autophagy in tumor cells. Despite studies on multiple cancer cell types, the clinical application of autophagy-targeted therapies remains challenging. High doses of CQ are required to achieve effective intratumoral concentrations, which may not be feasible in clinical settings. Moreover, CQ has been shown to activate secretory autophagy, leading to the secretion of IL-6 and IL-8, thereby contributing to treatment resistance [109]. Inhibiting the onset of autophagy represents a viable therapeutic strategy, but it is considerable that single inhibitor of autophagy not possess specificity. Therefore, based on the current research basis, we propose some theoretical directions.

### Establishing targeted therapy strategies for secretory autophagy

Novel targeted therapies for secretory autophagy require gradual establishment while considering the interplay of autophagy pathways. Bypassing cellular feedback and regulatory mechanisms is critical. Monoclonal antibodies targeting secretion-related proteins may provide effective options, though the cellular availability of monoclonal antibodies needs further evaluation. This challenge could be addressed by employing engineered nanotechnologies.

### Improvement of existing therapeutic drugs

Developing derivatives of existing drugs with enhanced bioavailability remains a key focus. A novel drug-loading strategy, the co-incubation method, involves incubating drugs with donor cells to induce new biological properties in exosomes by altering the culture conditions and incubation environment [358]. Engineered exosomes also offer promising potential for drug delivery [359]. For instance, exosomes derived from murine immature dendritic cells (IMDCs) have been used to mitigate immunogenicity and toxicity. Tumor-targeting capability was enhanced by binding the exosome membrane protein LAMP2B to an αv-integrin-specific iRGD peptide, creating a highly specific fusion peptide [360]. Furthermore, recent studies have demonstrated the feasibility of using mPEG-PLGA nanoparticles (DCV-NPs) for dehydrocurvularin drug delivery in breast cancer [361]. Advances in nanoengineering and cell engineering have also led to the development of hyaluronic acid (HA) nanogels co-loaded with CQ, facilitating intracellular delivery of cisplatin by targeting lysosomes [362].

Therapeutic strategies targeting secretory autophagy should be systematically evaluated both in vitro and in vivo. The advent of organoid and single-cell sequencing technologies offers a robust foundation for these efforts. Organoids, with their three-dimensional structures and preservation of in vivo molecular properties, provide new opportunities to assess molecular dynamics, pharmacokinetics, and pharmacodynamics [363–365]. Similarly, high-resolution single-cell sequencing enables precise experimental analyses, further advancing the field [366, 367].

## Data Availability

No datasets were generated or analysed during the current study.

## Abbreviations

3-MA: 3-methyladenine
ABCG2: ATP-binding cassette transporter G2
ACRs: Autophagy cargo receptors
ADAM10: A disintegrin and metalloprotease-10
ASS: Acute shear stress
ATGs: Autophagy related genes
ATM: Ataxia telangiectasia-mutated gene
BafA1: Bafilomycin A1
CAFs: Cancer associated fibroblasts
CASM: Conjugation of ATG8s to single membranes
CAV-1: Caveolin-1
CCA: Cholangiocarcinoma
CLEAR: Coordinated lysosomal expression and regulation
CRC: Colorectal cancer
CSC: Cancer stem cell
DFCP1: Double FYVE-containing protein 1
ER: Endoplasmic reticulum
ERK: Extracellular signal-regulated kinase
ESCC: Esophageal squamous cell carcinoma
EV: Extracellular vesicle
FAN: Fanconi anemia-associated nuclease
FIP200: Focal adhesion kinase family interacting protein of 200 kDa
GABARAP: GABA(A) receptor associated protein
GB: Glioblastoma
GIPC: GAIP-interacting protein C-terminus
GPR64: G-protein coupled receptor 64
GRASP: Golgi reassembly and stacking protein
HCC: Hepatocellular carcinoma
HDAC6: Histone deacetylase 6
HNSCC: Head and neck squamous cell carcinoma
HSP90AA1: Heat shock protein 90α family class A member 1
IKB: Inhibitor-kB
LC3: Microtubule-associated proteins 1 A/1B-light chain 3
LDELS: LC3-Dependent EV Loading and Secretion
LDHB: Lactate dehydrogenase B
MDR: Multidrug resistance
MIF: Migration inhibitory factor
mTORC1: Rapamycin complex 1
MVB: Multivesicular body
NF-kB: Nuclear factor kB
NFs: Normal fibroblasts
PDAC: Pancreatic ductal adenocarcinoma
PE: Phosphatidylethanolamine
PI3P: Phosphatidylinositol-3-phosphate
PSCs: Pancreatic stellate cells
PTEN: Phosphatase and tensin homolog
RAGE: Receptor for advanced glycation endproducts
RBP: RNA binding protein
SASP: Senescence-associated secretory phenotype
SCF: SKP1-Cullin 1-F-box protein
SFN: Sulforaphane
SKP1: S-phase Kinase-Associated Protein 1
SNARE: Soluble n-ethylmaleimide-sensitive factor attachment protein receptor
TCA: Tricarboxylic acid cycle
TFE3: Transcription factor that binds IGHM enhancer 3
TFEB: Transcription factor EB
THD: Thioridazine
TIMP1: Tissue inhibitor of metalloproteinase-1
TME: Tumour microenvironment
TMZ: Temozolomide
TRIM16: Tripartite motif-containing protein 16
ULK: Unc-51-like kinase
VAM: Vesicle associated membrane protein
VPS34: Vacuolar protein sorting 34
VPS34: Vacuolar protein-sorting 34
ZFYVE: Zinc finger containing FYVE domain
